# Sensitive Detection of Capsaicinoids Using a Surface Plasmon Resonance Sensor with Anti-Homovanillic Acid Polyclonal Antibodies

**DOI:** 10.3390/bios3040374

**Published:** 2013-11-13

**Authors:** Shingo Nakamura, Rui Yatabe, Takeshi Onodera, Kiyoshi Toko

**Affiliations:** Graduate School of Information Science and Electrical Engineering, Kyushu University, 744 Motooka, Nishi-ku, Fukuoka 819-0395, Japan; E-Mails: nakamura@belab.ed.kyushu-u.ac.jp (S.N.); yatabe@nbelab.ed.kyushu-u.ac.jp (R.Y.); toko@ed.kyushu-u.ac.jp (K.T.)

**Keywords:** capsaicinoids, surface plasmon resonance, immunosensor, homovanillic acid

## Abstract

Recently, highly functional biosensors have been developed in preparation for possible large-scale terrorist attacks using chemical warfare agents. Practically applicable sensors are required to have various abilities, such as high portability and operability, the capability of performing rapid and continuous measurement, as well as high sensitivity and selectivity. We developed the detection method of capsaicinoids, the main component of some lachrymators, using a surface plasmon resonance (SPR) immunosensor as an on-site detection sensor. Homovanillic acid, which has a vanillyl group similar to capsaicinoids such as capsaicin and dihydrocapsaicin, was bound to *Concholepas concholepas* hemocyanin (CCH) for use as an immunogen to generate polyclonal antibodies. An indirect competitive assay was carried out to detect capsaicinoids using SPR sensor chips on which different capsaicin analogues were immobilized. For the sensor chip on which 4-hydroxy-3-methoxybenzylamine hydrochloride was immobilized, a detection limit of 150 ppb was achieved. We found that the incubation time was not required and the detection can be completed in five minutes.

## 1. Introduction

Chemical warfare agents (CWAs) are chemical substances that, even in small quantities, rapidly act on humans to kill or incapacitate them. Large-scale terrorist attacks using CWAs (chemical terrorist attacks) may cause significant damage, and countermeasures based on scientific technologies are required. When chemical terrorist attacks occur, the causative substances must be detected and identified from the viewpoint of risk management and criminal investigation. To this end, the development of high-performance sensing systems that can be used to detect CWAs is required [1].

Lachrymators are CWAs that could be used in terrorist attacks. When people are exposed to a lachrymator, they develop various symptoms such as a burning sensation, pain, vomiting, and difficulty in breathing. Recently, self-defense (pepper) sprays have been frequently used in riots and minor offenses because of their high accessibility to the general public. The content of oleoresin capsicum (OC), which contains capsaicinoids extracted from *Capsicum*, is less than 10% in some self-defense sprays [2]. Capsaicin is normally found with dihydrocapsaicin in OC. Therefore, analytical methods of capsaicinoids are also required for on-site detection.

Studies on the analysis of capsaicin have been reported in several papers. They used gas chromatography mass spectroscopy (GC-MS) [2,3,4], high performance liquid chromatography [5], liquid chromatography mass spectroscopy (LC-MS) [6,7]. Limits of detection in the ppb range or lower ppm range for capsaicin were reported in those papers. Other methods were also reported, limit of detections for capsaicin using micellar electrokinetic capillary chromatography and using amperometric biosensor were found to be 1.75 ppm and 194 μM (*ca*. 0.6 ppm), respectively [8,9]. These methods basically require large stationary equipment in a laboratory, and experienced and skilled operators. There is, however, the risk that collected samples will be changed or disappear during transportation from the field to an analytical laboratory [1]. Kataoka *et al.* investigated detectability and stability of nonivamide, synthetic capsaicin, collected from the simulated situation for offensive odor incidents. The concentration of nonivamide was determined as 0.41 ng/μL (410 ppb) of the detection limit using GC-MS [10].

We have developed a sensing system using surface plasmon resonance (SPR) immunosensors with a compact size for on-site detection in terrorist attacks [11,12,13,14,15]. SPR immunosensors are realized by combining SPR sensors, which can be used to detect changes in the refractive index of the sensor surface with high sensitivity, with an antigen–antibody interaction, achieving high selectivity. The purpose of this study is to detect capsaicin and its related compounds, a main component of some lachrymators as a CWA, with high sensitivity using an SPR immunosensor. SPR immunosensor has potential to detect low molecular weight substances such as explosives, CWAs, and macromolecules, such as toxic proteins and viruses on the same platform. Thus, SPR immunosensors have great potential for use in on-site detection. 

In this study, we focused on the fact that capsaicin is found with other capsaicinoids, such as dihydrocapsaicin. Detection of capsaicin and dihydrocapsaicin, and their analogues in a lump is advantageous to the concentration of collected samples in the field for initial investigation. To recognize a vanillyl group of capsaicinoids in a lump, we prepared an original polyclonal antibody against homovanilic acid conjugated with *Concholepas concholepas* hemocyanin (CCH). We also fabricated a sensor chip on which capsaicin analogues are immobilized via a self-assembled monolayer (SAM) with oligo(ethylene glycol) chains. We chose an appropriate capsaicin analogue among three analogues for an indirect competitive assay. We needed to achieve 410 ppb of limit of detection (LOD) described above at least for on-site detection. The amount of capsaicinoids was measured by indirect competitive assay, with or without incubation time of mixing the antibody and capsaicinoid sample solution, using the SPR immunosensor we developed.

## 2. Experimental Section

### 2.1. Reagents

Capsaicin (C_18_H_27_NO_3_; molecular weight, 305.41; purity, 60%; rest 40% contains dihydrocapsaicin and derivatives; from capsicum; Wako Pure Chemical Industries, Ltd., Osaka, Japan), which contains dihydrocapsaicin and their analogue, was used as capsaicinoids to be measured. To obtain antibody, homovanillic acid (HVA; C_9_H_10_O_4_; molecular weight, 182.17; Sigma-Aldrich Corporation, St. Louis, MO, USA) was bound to *Concholepas concholepas* hemocyanin (CCH, Thermo Scientific Pierce, Rockford, IL, USA), and a rabbit was immunized with this immunogen. The obtained anti-HVA polyclonal antibody was used for the measurement of capsaicinoids. As capsaicin analogues, HVA, *N*-(3-hydroxy-4-methoxy-benzyl)-succinamic acid (NHM; C_12_H_15_NO_5_; molecular weight, 253.3; Otava Ltd., Ottawa, ON, Canada), and 4-hydroxy-3-methoxybenzylamine hydrochloride (HMB; C_8_H_11_NO_2_·HCl; molecular weight, 189.64; Wako Pure Chemical Industries, Ltd.) were used. Borate 8.5 (10 mM disodium tetraborate, 1 M NaCl, pH 8.5, GE Healthcare Bio-Science, Uppsala, Sweden) was used to dilute the capsaicin analogues. PEG6-COOH aromatic dialkanethiol (PEG6-COOH, SensoPath Technologies, Inc., Bozeman, MT, USA) was used as a reagent to form self-assembled monolayers (SAMs) on the chip surface. *N*-ethyl-*N*-(3-dimethylaminopropyl) carbodiimide hydrochloride (EDC) and *N*-hydroxysuccinimide (NHS) for sensor surface fabrication were purchased from GE Healthcare Bio-Science Ab. EDC and NHS for preparing immunogen were purchased from Nacalai Tesque, Inc. (Kyoto, Japan). The other reagents used were purchased from Tokyo Chemical Industry Co., Ltd. (Tokyo, Japan), Wako Pure Chemical Industries, Ltd., and Kanto Chemical Co., Inc. (Tokyo, Japan). All the reagents were of special grade. Water purified using a Milli-Q Integral Water Purification System (hereafter, Milli-Q water) was used as the solvent.

### 2.2. Preparation of Immunogen

HVA was prepared at a concentration of 2.1 mg/mL using *N*,*N*-dimethylformamide (DMF). NHS (4.7 mg) and sodium sulfate (102.6 mg) were added, and the mixture was stirred. EDC (67.5 mg) was then added and the mixture was stirred at room temperature overnight. 180 μL of this activated HVA solution was slowly added three times to CCH (50 mg) that had been dissolved in 12.5 mM borate buffer (1 mL, pH 8.0). After reaction for 3 h, the reaction solution was dialyzed against Milli-Q water overnight and freeze-dried to obtain HVA-CCH.

### 2.3. Preparation of Anti-HVA Polyclonal Antibody

Rabbit anti-HVA polyclonal antibodies were prepared by Genenet Co., Ltd. (Fukuoka, Japan), following a protocol lasting nine weeks, which involved five intradermal injections of immunogen into a rabbit. First, the immunogen prepared by the above procedure was added to Milli-Q water to produce a solution with a concentration of 0.3 mg/mL, which was emulsified using Freund’s complete adjuvant. The immunogen solution was intradermally injected into the rabbit on the 0th, 2nd, 4th, 6th, and 8th weeks. Blood was collected from the rabbit’s ear on the 5th and 7th weeks and used to determine the antibody titer by enzyme-linked immunosorbent assay (ELISA). Whole blood was collected on the 9th week, and the antiserum was purified by ion exchange chromatography and then dialyzed against 10 mM PB + 0.15 M NaCl (pH 7.2) to obtain anti-HVA polyclonal antibody.

### 2.4. Modification of Sensor Chip Surface

**Scheme 1:**
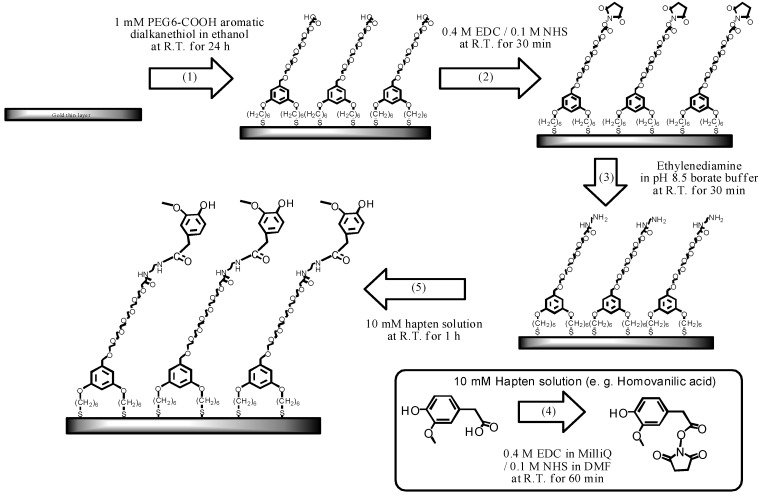
The fabrication process of the sensor chip modified with capsaicin analogue.

An Au sensor chip (SIA Kit Au, GE Healthcare Bio-Science Ab), the surface of which had not been modified, was used as the sensor chip. The surface of the sensor chip was modified following the procedure described in [16], as shown in Scheme 1.

As a preliminary cleaning step, the Au surface was first ultrasonically cleaned in acetone for 10 min, ethanol for 2 min, and 2-propanol for 2 min. Subsequently, the sensor surface was cleaned in standard clean (SC) 1 solution (mixture of ammonia solution, hydrogen peroxide solution, and pure water at a ratio of 1:1:5) heated to 90 °C for 20 min.

The preliminarily cleaned Au sensor surface was immersed in 1 mM PEG6-COOH ethanol for 24 h to form SAM on the sensor surface. After the sensor surface was ultrasonically cleaned in ethanol, the mixture solution (0.4 M EDC + 0.1 M NHS) was added dropwise onto the sensor surface and incubated for 30 min to activate the terminal carboxyl group. Next, ethylenediamine was added dropwise onto the sensor surface and incubated for 30 min to induce amine coupling. The terminal carboxyl group of the SAM was converted into an amino group. Simultaneously, a capsaicin analogue with a carboxyl group and the EDC + NHS solution were mixed and incubated for 60 min to activate the carboxyl group of the capsaicin analogue. The mixed solution was added dropwise onto the sensor surface and incubated for 60 min. Thus, the capsaicin analogue bound to ethylenediamine immobilized on the film surface as a result of amine coupling. Here, HMB has an amino group and does not require the introduction of an amino group onto the SAM; hence, HMB was added dropwise onto the sensor surface after the activation of the carboxyl group on the SAM. Blocking of active esters on the SAM with ethanolamine was not required in our studies [17].

### 2.5. Measurement Equipment

In the measurement, Biacore J (GE Healthcare Japan Corporation, Tokyo, Japan) was used as the SPR sensor. 10 mM 4-(2-hydroxyethyl)-1-piperazineethanesulfonic acid (HEPES) buffered saline (HBS, 150 mM NaCl, 0.05% Tween 20, pH 7.4) was used as the running buffer. The flow rate was 30 μL/min. The chip surface was regenerated (*i.e*., the antibodies were dissociated from the sensor surface) using 10 mM glycine-HCl (pH 1.5). The antibody solution was diluted using HBS. Capsaicin was dissolved in ethanol to obtain a capsaicinoids solution with a concentration of 10,000 ppm, which was diluted using HBS.

### 2.6. Measurement by Indirect Competitive Assay

In this study, the measurement was carried out by indirect competitive assay [18]. When the antibody solution is injected onto the chip surface, the antibodies bind to the capsaicin analogue immobilized onto the chip surface, resulting in an increased sensor response. The increase in the sensor response (Δ*θ*_0_) represents the amount of antibodies bound to the chip surface. For the SPR immunosensor used in this study, the sensor response is defined in resonance unit (RU), and 1000 RU is equivalent to an increase in the mass in a sensing matrix of about 1 ng/mm^2^ [17]. When the mixture of antibodies and capsaicinoids is injected onto the chip surface, the antibodies that have already bound to the capsaicinoids do not bind to the capsaicin analogue on the chip surface. Namely, the capsaicinoids in the solution inhibit the antibody from binding to the chip surface. Therefore, the concentration of antibodies bound to the chip surface decreases from that when only the antibodies are injected (Δ*θ_1_*). It is considered that Δ*θ*_1_ decreases as the percentage of capsaicinoids in the mixture increases. Here, the relative change in the concentration of antibodies bound to the chip surface, *i.e.*, the bound percentage, is calculated by

Δ*θ*_1_/Δ*θ*_0_ × 100% (1)

The smaller the bound percentage, the larger the concentration of antibodies bound to capsaicinoids.

The antibodies and capsaicinoids, each with a concentration twofold the final concentration, were prepared for the measurement. A mixture of the antibody solution and HBS buffer, whose concentrations were adjusted in accordance with the ethanol concentration of the capsaicinoid solution (mixing ratio, 1:1), was injected onto the chip surface for 5 min, followed by the injection of a regeneration solution for 3 min to regenerate the chip surface. Subsequently, the antibody and capsaicinoid solutions were mixed at a ratio of 1:1, and the resulting mixture was incubated for 30 min and similarly injected onto the chip surface for 5 min. Next, to regenerate the sensor surface, glycine-HCl solution was allowed to flow over the sensor surface for 3 min. To construct a calibration curve, the sensor response was measured three times each for capsaicinoid solutions with concentrations of 10 ppb, 100 ppb, 1 ppm, and 10 ppm following the above procedure. Finally, the sensor response was measured once for capsaicinoid solution with a concentration of 100 ppm, because capsaicinoids at high concentrations may adsorb to the flow channel, and subsequent cycles cannot be measured correctly. The bound percentage equal to a value threefold greater than the standard deviation was adopted as the detection limit.

### 2.7. Calculation of Association Constant

Antibody solutions with different concentrations (25, 50, 100, 200, 500, and 1,000 ppm) were injected onto the three sensor chips with different capsaicin analogues (HVA, NHM, and HMB) immobilized on the surface for 3 min at 30 μL/min. After the injection, the sensor chip was incubated for 3 min to allow spontaneous dissociation. The obtained sensorgram was used to calculate the reaction rate constants. BIAevaluation ver. 3.2 software (GE Healthcare Bio-Science) was used for analysis as well as the “bivalent with mass transfer” fitting model, which assumes that antibodies undergo bivalent binding and that the association of antibodies occurs under mass transport limitation. Six sensorgrams with six concentrations of the antibody solution were simultaneously analyzed by the fitting program to calculate the association rate constant *K_a_* and the dissociation rate constant *K_d_*. Using these constants, the association constant *K_A_* (=*K_a_*/*K_d_*) was calculated.

## 3. Results and Discussion

**Figure 1:**
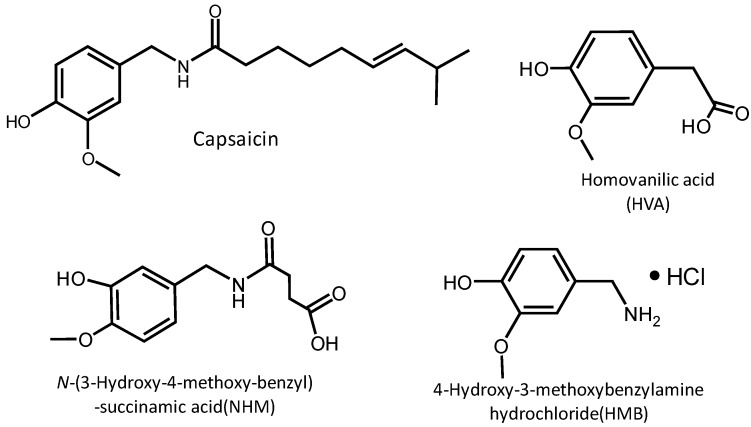
Structures of capsaicin and capsaicin-analogues.

Three sensor chips with different capsaicin analogues immobilized were prepared to detect capsaicinoids. Figure 1 shows the chemical structures of capsaicin and its analogues. The capsaicin analogues have a vanillyl group similar to capsaicin. It is known that oligo (ethylene glycol)-terminated SAM has a property which prevents nonspecific adsorptions of proteins [11,15,19]. When 100 ppm antibody solution was injected into the sensor chips, binding of the antibody to the three sensor chips was observed.

Next, a mixture of 50 ppm antibody solution (final concentration, 25 ppm) and capsaicinoid solution with a concentration of 200 ppb, 2 ppm, or 20 ppm (final concentration, 100 ppb, 1 ppm, or 10 ppm, respectively) was injected onto the HVA chip or HMB chip, and the change in the bound percentage was measured to examine the effect of capsaicinoids on inhibiting the antibodies from binding to the chip surface. For the NHM chip, capsaicinoid solutions with a concentration of 1,000 ppb, 10 ppm, or 100 ppm were used. 

**Figure 2:**
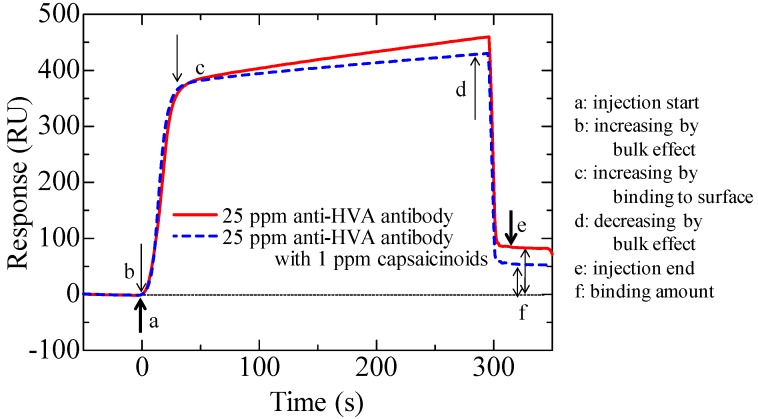
Surface plasmon resonance (SPR) sensorgrams of indirect competitive assay on HMB chip using 25 ppm anti-HVA polyclonal antibodies for detection of 1 ppm capsaicinoids.

Figure 2 shows a sensorgram obtained by this measurement on HMB chip. The sensor response sharply changes immediately after the start of the injection and immediately before the end of the injection because of the bulk effect (change in the refractive index of the solvent) caused by the injection of an ethanol-containing solution, which is not related to the binding of the antibody to the sensor surface. To adjust the effect of ethanol among the solutions with different capsaicinoids concentrations, ethanol was added to the reference antibody solution in accordance with the dilution rate of capsaicinoids so that each solution contained an equivalent concentration of ethanol. When a low-concentration target is subjected to indirect competitive assay, the concentration of antibodies in the solution becomes relatively high to target substances. The concentration may decrease the change in the bound percentage and therefore decrease the sensitivity. Hence, it is desirable to decrease the concentration of the antibody solution to as low as possible. For this reason, the final concentration of the antibody solution was set to 25 ppm, at which the sensor response of approximately 100 RU was obtained. 

Before injection onto the sensor surfaces, each mixture of the antibody and capsaicinoid solutions was incubated for 30 min to ensure that the antibodies and capsaicinoids react sufficiently. Table 1 shows the measurement results of the bound percentage for the three sensor chips. Each bound percentage shown in the table represents the mean of the values obtained in three measurements with standard deviation for each concentration. The 50 ppm capsaicinoids on NHM chip was measured once. The bound percentage for the HMB immobilized chip decreased with increasing capsaicinoid concentration, whereas the bound percentages for the chips with immobilized NHM or HVA remained almost the same as those when only the antibody solution was injected. 

**Table 1:** Bound percentage with standard deviation for immobilized capsaicin analogues with indirect competitive assay for detection of capsaicinoids.

	Bound (%)					
Capsaicin analogues	Capsaicinoids					
	**100 ppb**	**500 ppb**	**1 ppm**	**5 ppm**	**10 ppm**	**50 ppm**
HVA	100.6 ± 1.8		94.2 ± 0.5		94.2 ± 1.4	
NHM		93.1 ± 0.7		100.4 ± 1.4		96.8
HMB	97.8 ± 1.1		68.5 ± 1.0		42.6 ± 9.3	

The antibodies used in this study were obtained using an HVA-CCH conjugate antigen as the immunogen. Reduction of binding antibody to HVA was observed on all three chips (see Appendix). However, the reduction of bound percentage due to the existence of capsaicinoids was only observed on the HMB chip. The bound percentage at 10 ppm HVA was higher than that at 10 ppm capsaicinoids on the HMB chip. 

NHM and HMB have a similar structure to HVA, therefore, the association constant of the antibodies to these three capsaicin analogues-immobilized sensor chips was analyzed. From the calculation results, *K_A_* decreased in the order NHM (2.90 × 10^4^ M^−1^) > HVA (1.80 × 10^4^ M^−1^) > HMB (1.17 × 10^4^ M^−1^). *K_A_* for the HMB chip, which successfully detected capsaicinoids, was smaller than those for the HVA or NHM chips. There is a possibility that antibodies having slightly different antigen-binding site existed in the polyclonal antibody. NHM has a short side chain with amide bond, and positions of the methoxy group and hydroxyl group differ between HVA and HMB. However, *K_A_* on NHM chip was larger than on the others. It may well be that antibodies can interact with NHM and cannot interact with capsaicinoids. 

The difference in the structures of HVA and HMB is the order of carbonyl and amine in the amide bond. The antibody rose against the HVA-CCH conjugate; therefore the affinity to HVA was stronger than HMB. Vanillin, which has an aldehyde group, was also measured from 10 ppm to 1,000 ppm on the HMB chip by indirect competitive assay. However, only 10% reduction of bound percentage was observed at 100 ppm (data not shown). This result suggests that the existence of a benzyl group in the target substance is important for recognition of the target substances with this anti-HVA polyclonal antibody on HMB chip. These results indicate that the HMB chip is most appropriate for detecting capsaicinoids among the three sensor chips examined in this study.

We examined the effect of incubation time on detection for rapid measurement. To evaluate the effect of incubation time, the antibody and capsaicinoid solutions (1 ppm) were mixed, and the resulting mixture was incubated for 0, 5, 15, 30, or 60 min and injected onto the HMB chip surface. The bound percentage was measured for each incubation time once. The other measurement conditions were the same as in Section 2.6. Table 2 shows the measurement results. The bound percentages were not dependent on incubation times, indicating that incubation time does not affect sensitivity in this measurement.

**Figure 3:**
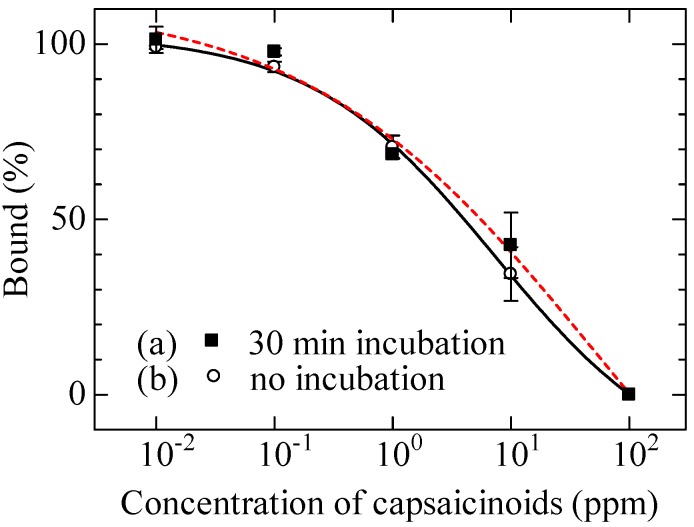
Response characteristic to capsaicinoids on HMB modified chip.

We constructed a calibration curve by carrying out an experiment on capsaicinoid detection using the sensor chip on which HMB was immobilized. Figure 3(a) shows the average with standard deviation of the three measurements. As shown in the figure, the bound percentage decreased with increasing capsaicinoid concentration. This is because the binding of the antibodies to the chip surface was inhibited by capsaicinoids, meaning that this sensor chip successfully detected capsaicinoids. The detection limit was 150 ppb in this measurement.

Considering the results in Table 2, we constructed a calibration curve in the case without incubation for shortening measurement time. The result is shown in Figure 3(b). Even without incubation, a calibration curve similar to that obtained in the case of 30 min incubation was obtained, and a detection limit of 150 ppb was similarly achieved.

**Table 2:** Bound percentage of 25 ppm anti-HVA antibodies with 1 ppm capsaicinoids for each incubation time.

Bound (%)				
Incubation time				
0 min	5 min	15 min	30 min	60 min
74.9	80.4	79.6	85.8	81.4

## 4. Conclusions

We developed a sensitive method for detecting capsaicinoids using an SPR immunosensor for on-site detection of lachrymator, which is a chemical warfare agent. We prepared rabbit-derived anti-HVA polyclonal antibody and measured capsaicinoids by an indirect competitive assay, using sensor chips with different capsaicin analogues immobilized on SPR sensor surface via oligo (ethylene glycol)-terminated SAM. When a calibration curve was constructed using the sensor chip with HMB immobilized, a detection limit of 150 ppb was achieved with an antibody concentration of 25 ppm. We also evaluated the effect of incubation time and demonstrated that, even without incubation, capsaicinoid detection was possible within five minutes at an accuracy equivalent to that detected with incubation.

## Figures and Tables

**Figure S1:**
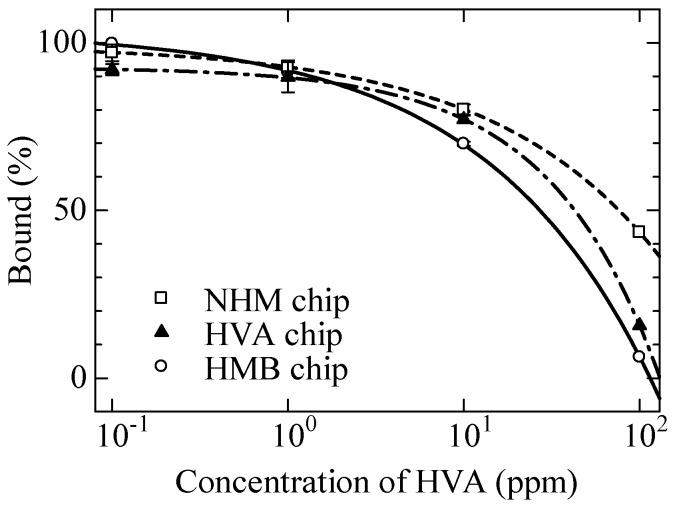
Response characteristic to homovanillic acid on NHM, HVA, and HMB modified chips.

## Appendix

We constructed a calibration curve to HVA by indirect competitive assay using the three sensor chips. The experiments were carried out in the same way as used for capsaisinoid measurements. The results are shown in Figure S1. A reduction of bound percentage was observed on all three sensor chips.
